# Differential motor signatures in isolated and narcolepsy-related REM sleep behaviour disorder: a preliminary study

**DOI:** 10.3389/fneur.2025.1749306

**Published:** 2026-01-14

**Authors:** Panagis Drakatos, Iain Duncan, Danielle Wasserman, Valentina Gnoni, David O’Regan, Andrew W. Varga, Huiling Tan, Keyoumars Ashkan, Lea T. Grinberg, Liborio Parrino, Luigi Ferini-Strambi, Peter J. Goadsby, K. Ray Chaudhuri, Ivana Rosenzweig

**Affiliations:** 1 Sleep Disorders Centre, Guy’s and St Thomas’ Hospital (GSTT NHS), London, United Kingdom; 2 Faculty of Life Sciences and Medicine, King’s College London, London, United Kingdom; 3 Sleep and Brain Plasticity Centre, Department of Neuroimaging, Institute of Psychiatry, Psychology and Neuroscience (IoPPN), King’s College London, London, United Kingdom; 4 Mount Sinai Integrative Sleep Center, Division of Pulmonary, Critical Care, and Sleep Medicine, Icahn School of Medicine at Mount Sinai, New York, NY, United States; 5 MRC Brain Network Dynamics Unit, Nuffield Department of Clinical Neurosciences, University of Oxford, Oxford, United Kingdom; 6 Department of Neurosurgery, King’s College Hospital Foundation Trust, London, United Kingdom; 7 Department of Laboratory Medicine and Pathology, Mayo Clinic, Jacksonville, FL, United States; 8 Sleep Disorders Centre, Parma University Hospital, Parma, Italy; 9 Sleep Disorders Center, Università Vita-Salute San Raffaele, Milan, Italy; 10 NIHR-Wellcome Trust King’s Clinical Research Facility, King’s College London, London, United Kingdom; 11 King’s College London, London, United Kingdom

**Keywords:** isolated RBD, motor semiology, narcolepsy type 1, neurodegeneration, orexin, REM sleep behaviour disorder, sleep neurophysiology, video-polysomnography

## Abstract

**Background:**

REM sleep behaviour disorder (RBD) is a prodrome of *α*-synucleinopathy, yet mechanistic pathways are unresolved. Narcolepsy type 1 with RBD (NT1-RBD) provides a human model of orexin deficiency. We tested whether REM motor semiology differs categorically between isolated RBD (iRBD) and NT1-RBD.

**Methods:**

We retrospectively analyzed blinded video-polysomnographic scorings from 57 patients (iRBD *n* = 34; NT1-RBD *n* = 23). Across 857 REM events (iRBD 717; NT1-RBD 140), we classified topography (head/neck, trunk, upper, lower limbs), complexity (elementary vs. complex), content (scenic, violent, self-referential), vocal/orofacial features, spatial distribution and laterality using a pre-specified codebook. The patient was the primary unit of inference. Binary features used Fisher’s exact tests with Cohen’s *h*; the per-patient complex-event proportion used Mann–Whitney and Cliff’s *δ* (bootstrap 95% CI). Robustness checks comprised Beta-Binomial posteriors (Beta [1,1]) and patient-label permutation tests (10,000 permutations).

**Results:**

REM motor phenotypes diverged categorically. Lower-limb dominance occurred in 18/23 (78.3%) NT1-RBD vs. 7/34 (20.6%) iRBD (Fisher *p* < 0.0001; Cohen’s *h* ≈ +1.23), whereas upper-limb dominance occurred in 24/34 (70.6%) iRBD vs. 2/23 (8.7%) NT1-RBD (*p* < 0.0001; *h* ≈ −1.40). Any complex event was present in 27/34 (79.4%) iRBD vs. 3/23 (13.0%) NT1-RBD (*p* < 0.0001; *h* ≈ −1.46); violent enactments in 16/34 (47.1%) vs. 0/23 (0%) (*p* < 0.0001; *h* ≈ −1.51). The per-patient complex-event proportion was higher in iRBD [median 0.21 (0.05–0.33)] than NT1-RBD [0.00 (0.00–0.00)] (Mann–Whitney *p* < 0.0001; Cliff’s *δ* = −0.665; 95% CI −0.853 to −0.441). Event-level summaries were concordant; permutation *p*-values were 0.0001 for upper-limb involvement and 0.0083 for complex behaviour.

**Conclusion:**

iRBD and NT1-RBD exhibit qualitatively distinct REM motor phenotypes: upper-body-dominant, complex/scenic behaviours in iRBD versus elementary, predominantly bilateral lower-limb behaviours with notable trunk recruitment in NT1-RBD, supported by large effect sizes and convergent robustness checks. These findings motivate mechanistic studies of hypothalamic-brainstem-cortical integration in REM and suggest that semiological profiling may aid stratification in prodromal neurodegeneration.

## 1 Introduction

Parkinson disease (PD) is the fastest growing neurological disorder and a leading source of disability globally (1). Among its earliest non-motor features is isolated REM sleep behaviour disorder (iRBD), a parasomnia defined by loss of REM-associated atonia and complex dream enactment behaviours (2, 3). iRBD often precedes overt alpha-synucleinopathy by years and carries a high risk of phenoconversion to PD or dementia with Lewy bodies (4). Yet, despite its predictive value, mechanistic understanding of RBD remains limited, and effective, pathophysiology-informed treatments are lacking.

Patients with iRBD typically report vivid, aversive dream content involving pursuit or confrontation, often accompanied by motor behaviours that mirror these narratives (5). These enactments arise predominantly during phasic REM sleep, a microstate characterized by rapid eye movements, motor bursts, and cortical activation (6). Prior work suggests that iRBD movements are not random but reflect structured, contextually embedded simulations of defensive actions within a brain-generated allocentric space (5, 7, 8). These simulations often engage upper limbs and head/neck musculature, but typically do not result in bed exit, suggesting internalized action schemas (8).

The mechanistic substrate of this motor expressivity is unresolved. Recent preclinical studies have implicated orexinergic modulation in REM motor control (9). Orexin (hypocretin), a neuropeptide produced in the lateral hypothalamus, is classically known for its role in arousal, sleep–wake transitions, and energy regulation (10, 11). However, orexin also projects widely to brainstem, limbic, and cortical targets involved in motor preparation and affective processing (10, 11). Although orexin’s precise role in RBD remains debated (12), the presence of REM without atonia in both iRBD and NT1 has prompted competing hypotheses, ranging from hyperactivity to complete loss of orexin tone (13–15). Dual orexin receptor antagonists (DORAs) have shown efficacy in suppressing REM-associated behaviours in models of tauopathy (9), raising the possibility that orexin modulates not only REM atonia but also REM motor content.

**Figure 1:**
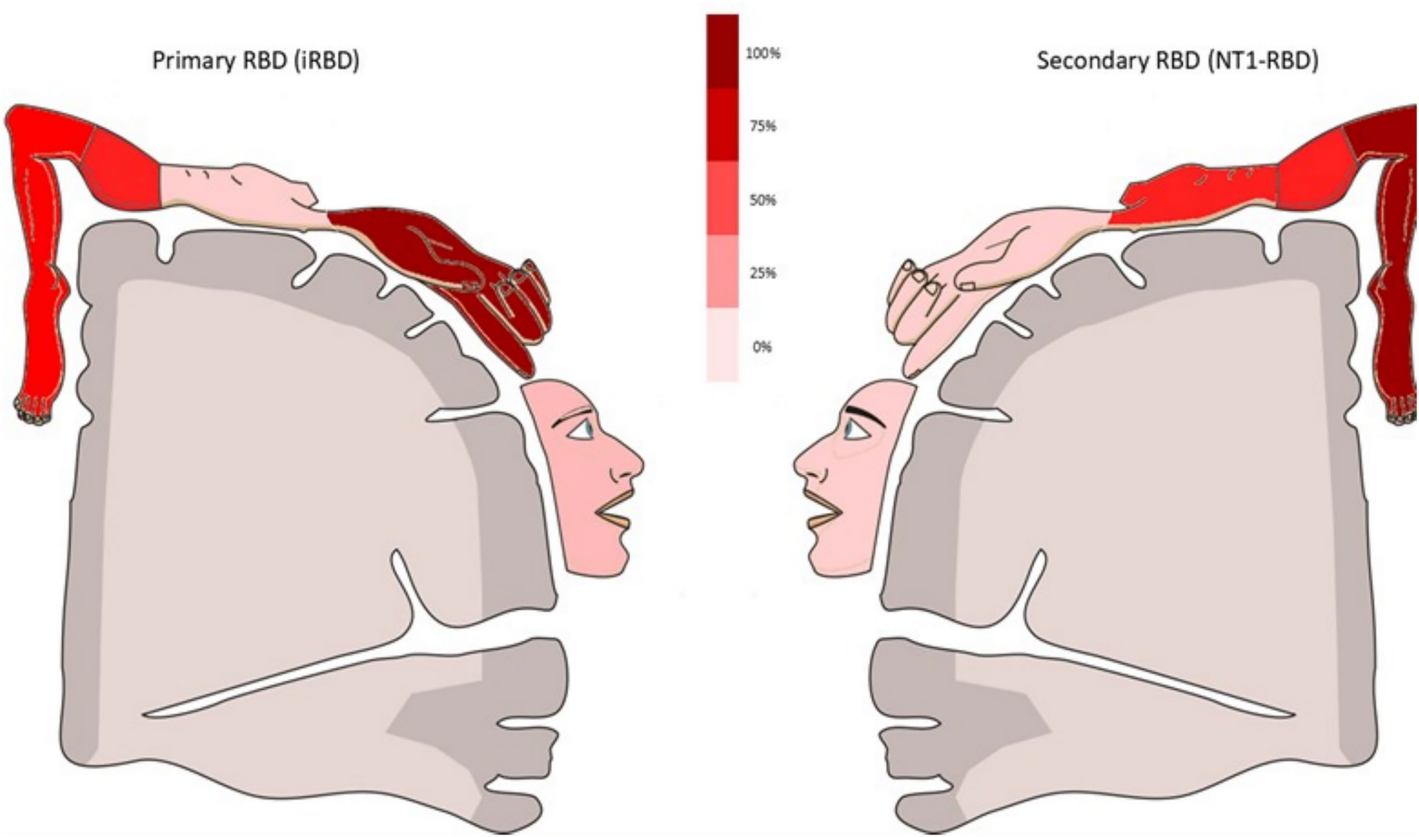
Illustrative summary of relative body-region involvement during REM enactments in iRBD and NT1-RBD. Schematic depiction of the relative frequency of event-level body-region involvement in isolated RBD (iRBD) and narcolepsy type 1-associated RBD (NT1-RBD), with color intensity scaled to within-group percentages (scale bar). Quantitative event-level denominators and proportions are reported in Supplementary Tables S2, S3; direct between-group comparison of event-level topography is shown in Figure 4.

**Figure 2:**
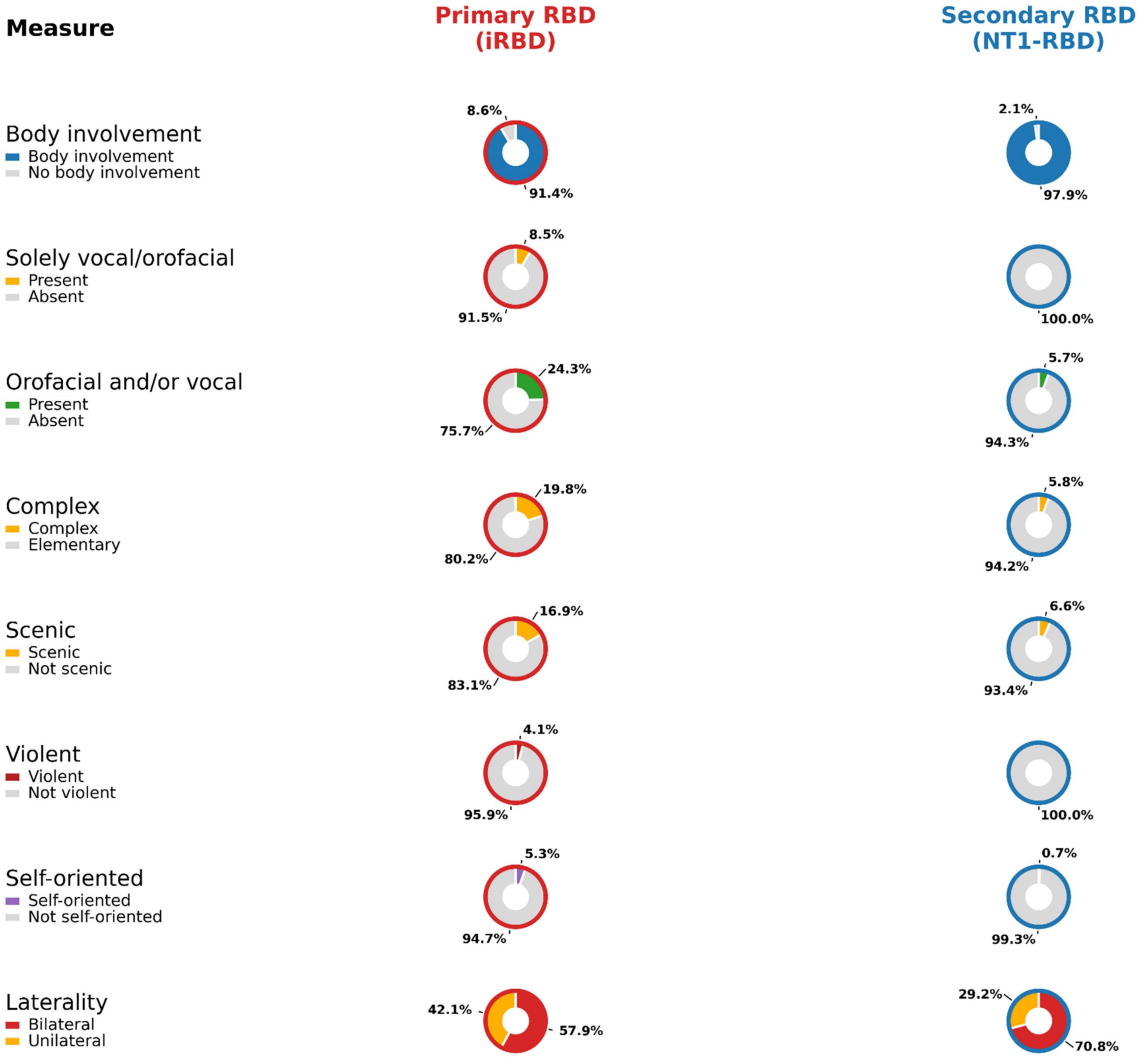
Direct event-level comparison of REM sleep behaviour disorder (RBD) semiology in isolated RBD and narcolepsy type 1-associated RBD. Paired donut plots show, for each semiological feature, the proportion of scored REM enactment events meeting the definition in isolated RBD (iRBD; left) and narcolepsy type 1 with comorbid RBD (NT1-RBD; right). Percentages are displayed outside each ring; segment colors correspond to the per-row keys. Denominators follow the prespecified codebook (Supplementary Table S9) and event-corpus totals (Supplementary Table S2). Specifically, body involvement, solely vocal/orofacial events, and orofacial and/or vocal features (any) use all scored REM events as denominator; complexity, scenic content, violent content, and self-oriented behaviours use body-involvement events as denominator. Laterality summarises the distribution of bilateral versus unilateral movements among events for which laterality was scored. iRBD, isolated REM sleep behaviour disorder; NT1, narcolepsy type 1; NT1-RBD, narcolepsy type 1-associated RBD; RBD, REM sleep behaviour disorder; REM, rapid eye movement.

Narcolepsy type 1 (NT1), a chronic disorder marked by near-complete loss of orexin-producing neurons, presents a natural model for REM sleep dysregulation (15). While REM sleep without atonia is common in NT1, the qualitative features of dream enactment have received less attention (15). Up to 60% of NT1 patients meet criteria for secondary RBD, yet available evidence suggests their REM behaviours are simpler, less emotional, and more stereotyped than those in iRBD (15). Whether this reflects orexin deficiency per se, or a broader neurochemical distinction, remains unclear. To address this, we retrospectively analyzed video-polysomnography (VPSG) data from 57 patients diagnosed over a two year period at a UK tertiary sleep disorders centre: 34 with iRBD and 23 with NT1 and comorbid RBD (NT1-RBD) (Figures 1, 2). Diagnoses were based on American Academy of Sleep Medicine (AASM) criteria (ICSD-3; AASM Manual v2.4) (16, 17), and all patients underwent standardized VPSG with synchronized video, EEG, EMG, and respiratory channels.

## 2 Methods

### 2.1 Study design and oversight

We conducted a retrospective, observational study at a UK tertiary sleep-disorders centre, reported in accordance with STROBE guidance. Consecutive patients diagnosed over a 24-month interval with isolated REM sleep behaviour disorder (iRBD) or narcolepsy type 1 with comorbid RBD (NT1-RBD) were screened from clinical records and video-polysomnography (VPSG). Ethical approval was granted by the Hospital Clinic Research Ethics Committee (GSTT NHS, UK; Project No. 9585). All data were fully anonymised and handled in accordance with the Declaration of Helsinki and GDPR.

Fifty-seven VPSGs were screened. The analytic semiology cohort comprised 57 patients with analyzable video and scorable REM motor events: 34 iRBD and 23 NT1-RBD. All semiological analyses (main text and Supplementary material) refer to this analytic cohort. At the event level, the corpus comprised 857 scored REM events (iRBD 717; NT1-RBD 140), of which 792 involved the body (iRBD 655; NT1-RBD 137). These denominators are used consistently throughout and are explicitly stated in the Supplementary material. Diagnoses were established by subspecialist clinicians using International Classification of Sleep Disorders criteria and American Academy of Sleep Medicine (AASM) criteria (ICSD-3; AASM Manual v2.4) (16). NT1 was diagnosed by history, Multiple Sleep Latency Test and/or CSF orexin when available; iRBD required clinical history consistent with RBD and REM sleep without atonia on VPSG with corroborated enactment.

### 2.2 Video-polysomnography acquisition

Overnight VPSG was recorded with synchronized infrared video (and audio where available) using a standard clinical montage, as previously reported (8). Signals included scalp EEG using a 10–20 montage (frontal, central and occipital derivations, referenced to contralateral mastoids), bilateral EOG, single-lead ECG, submental EMG, and bilateral tibialis anterior EMG. Standard respiratory channels were recorded (nasal pressure and/or oro-nasal thermistor, thoraco-abdominal effort belts, pulse oximetry), together with snore and body-position where available as part of routine clinical PSG. Sleep staging followed AASM rules (16).

### 2.3 Event identification and segmentation

Candidate REM motor episodes were identified visually and confirmed on EMG, as previously described (8, 18). Events were included if they (i) occurred during REM sleep, (ii) lasted ≥2 s, and (iii) were not temporally adjacent to respiratory events or EEG arousals (*a priori* exclusion to minimise confounding by sleep-disordered breathing or periodic limb movements).

### 2.4 Periodic limb movements

Periodic limb movements (PLMs) during sleep were scored as part of the routine clinical polysomnography report but were not available in a consistent fashion for the final NT1-RBD cohort. In the iRBD cohort, PLM indices were available in a subset and are reported descriptively in Supplementary Table S1. We did not compare PLMI formally between groups, and we mitigated potential PLM contamination by (i) excluding events temporally adjacent to arousals or respiratory events, (ii) requiring visible video movement for limb topography, and (iii) using the patient as the primary unit of inference.

Distinct events were separated by ≥2 s of quiescence with EMG returning to baseline and no visible movement on video. Event clock-time and REM-cycle position were not retained as structured variables in this retrospective scoring database; therefore, we did not analyse within-night temporal distribution of events. A brief post-sleep questionnaire was administered as part of clinical practice; where available, observed enactments were cross-checked against dream recall, as previously published (19, 20).

### 2.5 Semiology taxonomy and operational definitions

Each event was classified using a pre-specified codebook adapted from published frameworks (8, 18). Categories comprised: (a) topography (head/neck, trunk, upper limbs, lower limbs); (b) complexity (elementary vs. complex); (c) qualifiers (scenic, violent, self-referential); (d) vocal/orofacial features; (e) spatial distribution (focal vs. multifocal); and (f) laterality (unilateral vs. bilateral). Topographical categories are non-exclusive and may co-occur within an event; consequently, their percentages are computed within the relevant event denominator and may sum to >100%. For certain analyses (complexity, scenic, violent, self-referential), the denominator was restricted to body-involvement events (i.e., events with visible body movement), whereas topography and orofacial/vocal features used all events as the denominator. These denominator rules are stated in table footnotes and applied uniformly. Two experienced raters, blinded to diagnosis, scored all recordings independently using the codebook. Discrepancies were resolved by consensus in a joint session. On a randomly selected subset of 20 patients, double-scoring yielded substantial agreement for body-region and complexity (Cohen’s *κ* = 0.82 and 0.79, respectively).

### 2.6 Derived patient-level features

The primary unit of analysis was the patient. For each patient, we derived binary “ever” features (any upper-, lower-, trunk-, head/neck-involvement; any complex/scenic/violent/self-referential; any vocal/orofacial; any bilateral lower-limb) and a continuous per-patient proportion of complex events (complex/body-involvement). Dominant region was defined as the most frequently involved body region across a patient’s events; ties were rare and adjudicated by consensus before data-lock.

### 2.7 Statistical analysis

Descriptive statistics are reported as *n* (%), median (IQR), or mean ± SD. For patient-level binary features we used Fisher’s exact tests with Cohen’s *h* as effect size; for the per-patient complex proportion we used Mann–Whitney *U* with Cliff’s *δ* (bootstrap 95% CI, 5,000 resamples). Event-level summaries are descriptive (counts, proportions, Cohen’s *h*) with explicit denominators; no event-level *p*-values are reported due to clustering.

### 2.8 Sensitivity analyses

To bound potential confounding and examine opportunity bias, we conducted three patient-level sensitivity analyses using the same scoring rules and denominators as the primary analysis. (i) Antidepressant-negative subset: exposure was defined per patient (exposed if any event in that patient’s recording carried an antidepressant flag at the time of VPSG); analyses were repeated after excluding exposed patients. (ii) Age-restricted contrast: because there is no age overlap between groups, we contrasted the 12 youngest iRBD patients with the full NT1-RBD cohort (acknowledging residual age differences). (iii) Sex-restricted contrast: we repeated analyses in male-only patients (female-only results are provided descriptively in the Supplementary material given the very small iRBD female *n*). In each subset we recomputed the patient-level “ever” features (dominant region; any upper/lower/trunk/head-and-neck involvement; any complex, scenic, violent, self-referential; any orofacial/vocal; any bilateral lower-limb) using the same denominator rules (topography/orofacial-vocal: all events; complexity/content: body-involvement events) and the per-patient complex-event proportion (complex/body-involvement). Statistical summaries parallel the primary analysis: Fisher’s exact tests with Cohen’s *h* for binary features; Mann–Whitney *U* with Cliff’s *δ* (bootstrap 95% CI) for the per-patient proportion. Given reduced sample sizes and persistent non-overlap, *p*-values are descriptive and we emphasise direction and effect-size magnitude; no multiplicity correction was applied. Permutation tests (described above) assess event-level separation under patient-label exchangeability and were not re-run within these subsets. Full outputs appear in the Supplementary Table S8 (sex splits) and Supplementary Sensitivity-Matched Subsets appendix.

Robustness checks comprised patient-label permutation tests (10,000 permutations; labels shuffled at the patient level, preserving within-patient clustering); event-level differences were recomputed each time; two-sided empirical *p* is the fraction of permuted differences ≥the observed. Given marked demographic non-overlap and perfect/near-perfect separation in several features, conventional adjusted logistic regression was not pursued for primary inference; penalised/Bayesian logistic models are reserved for sensitivity only.

### 2.9 Reproducibility and denominators

All tables state their denominator rule (all events vs. body-involvement). Topographical categories are non-exclusive. Event-adjacency exclusions were applied *a priori*. Full contingency tables, denominators and effect sizes are provided in the Supplementary Tables S1–S9 and Supplementary Figures S1–S6.

## 3 Results

### 3.1 Participants and recordings

Fifty-seven video-polysomnographies (VPSGs) met inclusion (isolated RBD, iRBD *n* = 34; narcolepsy type 1 with RBD, NT1-RBD *n* = 23). We scored 857 REM events (iRBD 717; NT1-RBD 140), of which 792 involved visible body movement (iRBD 655; NT1-RBD 137). Denominators are used consistently across tables and figures (Supplementary Tables S2–S8 and Supplementary Figures S1a,b, S2–S5). Periodic limb movement indices were available only for a subset of iRBD patients and are reported descriptively in Supplementary Table S1; PLMI was not systematically recorded for the NT1-RBD cohort in this epoch. PLMI was available for 33/34 iRBD patients (mean 28.8 ± 40.4 events/h) but was not systematically scored for NT1-RBD in this clinical epoch (Supplementary Table S1), precluding a balanced between-group PLMI sensitivity analysis.

### 3.2 Sleep architecture

Conventional PSG architecture differed between groups in expected ways (Supplementary Table S1). NT1-RBD had longer total sleep time (433.5 ± 94.7 vs. 326.2 ± 54.0 min, *p* < 0.0001), higher sleep efficiency (84.5 ± 12.5 vs. 70.9 ± 13.2%, *p* < 0.0001), and less wake after sleep onset (71.4 ± 62.9 vs. 108.6 ± 61.0 min, *p* = 0.0064). REM sleep latency was markedly shorter in NT1-RBD (37.4 ± 59.7 vs. 148.9 ± 111.0 min, *p* < 0.0001). Importantly, REM sleep percentage did not differ significantly between groups (22.10 ± 6.48% vs. 19.31 ± 6.14%, *p* = 0.1004). Using REM latency ≤15 min as a PSG SOREMP definition, SOREMPs were present in 13/23 (56.5%) NT1-RBD and 0/34 (0%) iRBD (Fisher *p* < 0.000001).

### 3.3 Primary patient-level analyses

Semiological features showed categorical separation between groups with large effect sizes. Lower-limb dominance occurred in 18/23 (78.3%) NT1-RBD versus 7/34 (20.6%) iRBD (Fisher *p* < 0.0001; Cohen’s *h* ≈ +1.23), whereas upper-limb dominance occurred in 24/34 (70.6%) iRBD versus 2/23 (8.7%) NT1-RBD (*p* < 0.0001; *h* ≈ −1.40). Complex, affectively salient behaviours were concentrated in iRBD: any complex event in 27/34 (79.4%) iRBD versus 3/23 (13.0%) NT1-RBD (*p* < 0.0001; *h* ≈ −1.46); violent enactments in 16/34 (47.1%) versus 0/23 (0.0%) (*p* < 0.0001; *h* ≈ −1.51); scenic in 23/34 (67.6%) versus 3/23 (13.0%) (*p* < 0.0001; *h* ≈ −1.19); self-referential in 13/34 (38.2%) versus 1/23 (4.3%) (*p* = 0.0041; *h* ≈ −0.91). Upper-limb involvement (ever) was seen in 33/34 (97.1%) iRBD versus 11/23 (47.8%) NT1-RBD (*p* < 0.0001), and head/neck involvement in 26/34 (76.5%) versus 9/23 (39.1%) (*p* = 0.0062). Bilateral lower-limb involvement was common in both groups [29/34 (85.3%) iRBD; 20/23 (87.0%) NT1-RBD; *p* = 1.0000]. Full patient-level counts and effect sizes are tabulated in Supplementary Table S6.

### 3.4 Per-patient proportion of complex events

The proportion of complex events per patient (denominator: body-involvement events) was markedly higher in iRBD (median 0.21, IQR 0.05–0.33) than NT1-RBD (0.00, 0.00–0.00) (Mann–Whitney *p* < 0.0001; Cliff’s *δ* = −0.665, bootstrap 95% CI −0.853 to −0.441) (see Supplementary Table S7 and Supplementary Figure S2).

**Figure 3:**
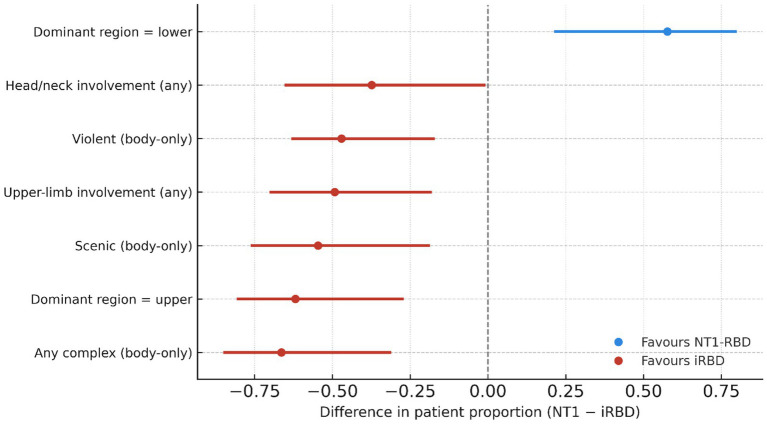
Patient-level differences in semiological features between secondary [NT1-RBD (*n* = 23)] and primary [iRBD (*n* = 34)] RBD. Points represent the difference in patient proportions (NT1 − iRBD) with Newcombe (Wilson) 95% confidence intervals. Topographical “dominant region” values were derived per patient from all scored events, whereas content and complexity features were computed from body-involvement events only, mirroring denominators in Supplementary Table S6. Positive values favor NT1-RBD; negative values favor iRBD. This forest plot complements Figures 2, 4 by quantifying the categorical separation (NT1 − iRBD) with 95% CIs.

A compact patient-level forest plot (Figure 3) quantifies these differences as proportion contrasts with 95% CIs (NT1 minus iRBD), highlighting NT1’s lower-limb dominance and iRBD’s enrichment of upper-limb, complex, scenic, and violent behaviours.

### 3.5 Event opportunities and denominators

Because iRBD patients contributed more scored REM events per patient than NT1-RBD, “ever” outcomes can reflect opportunity bias. In the analytic cohort, the number of scored REM events per patient was 20 [8–28] in iRBD versus 5 [3–8] in NT1-RBD; restricting to body-involvement events, 19 [8–25] versus 4 [3–8] [medians (IQR), rounded to integers]. We therefore pre-specified the per-patient complex-event proportion as the primary patient-level measure and present “ever” features as secondary descriptors.

### 3.6 Event-level characterisation (descriptive)

**Figure 4:**
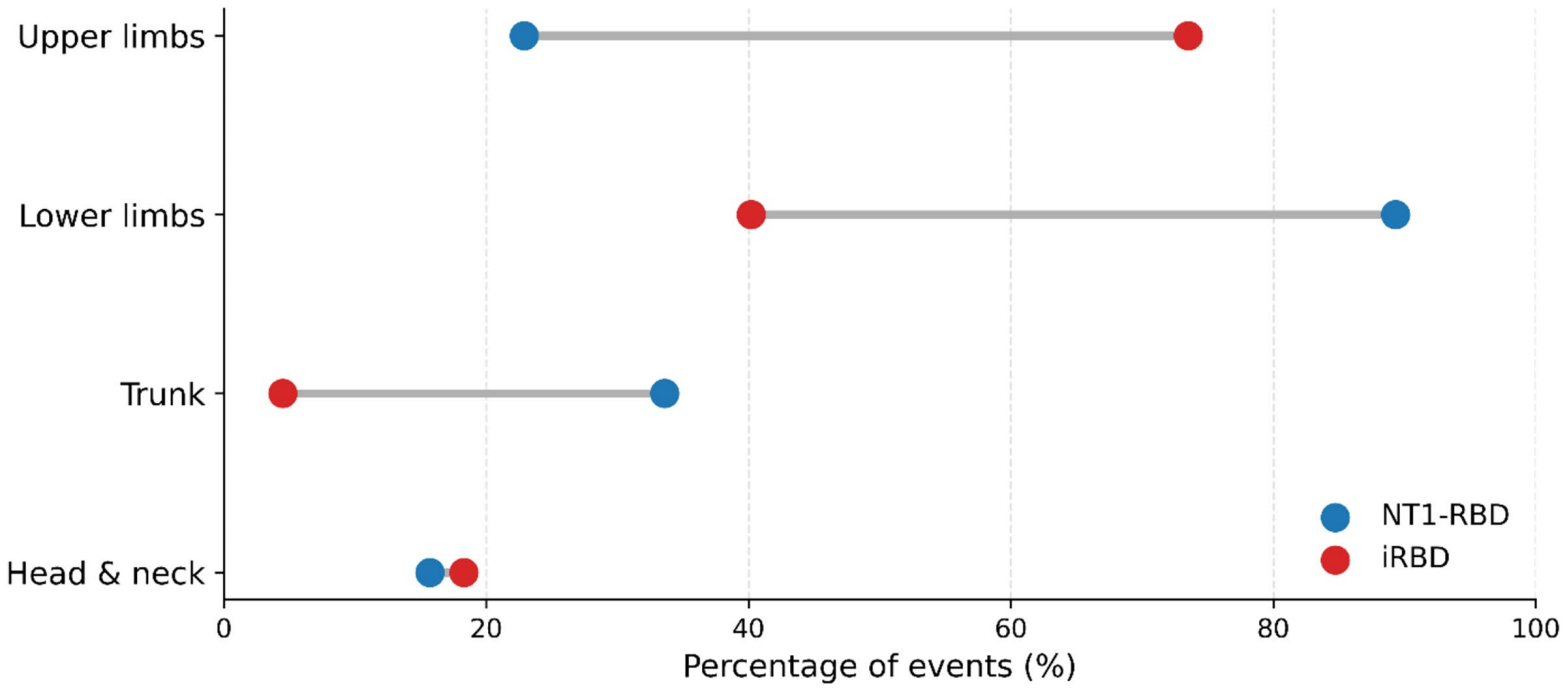
Event-level topography in NT1-RBD versus iRBD. Dumbbell plot showing the percentage of all scored REM sleep behaviour disorder events involving each body region in NT1-RBD (blue) and iRBD (red). Gray connectors indicate the between-group separation for each region. Percentages are calculated using all scored events as the denominator; topographical categories are non-exclusive, so totals may exceed 100%. Denominator: all scored REM events (iRBD *n* = 717; NT1-RBD *n* = 140).

Event-level summaries (counts, proportions, and Cohen’s *h*; no event-level *p*-values due to clustering) were congruent with patient-level dissociation. With all events as denominator, upper-limb involvement was 527/717 (73.5%) in iRBD versus 32/140 (22.9%) in NT1-RBD (Cohen’s *h* = −1.06); lower-limb involvement 288/717 (40.2%) versus 125/140 (89.3%) (*h* = +1.10); trunk 32/717 (4.5%) versus 47/140 (33.6%) (*h* = +0.81); head/neck 131/717 (18.3%) versus 22/140 (15.7%) (*h* = −0.07). Restricting the denominator to body-involvement events, complex behaviour occurred in 130/655 (19.8%) iRBD versus 8/137 (5.8%) NT1-RBD (*h* = −0.44); elementary behaviour in 525/655 (80.2%) versus 129/137 (94.2%) (*h* = +0.44); scenic 111/655 (16.9%) versus 9/137 (6.6%) (*h* = −0.33); self-oriented 35/655 (5.3%) versus 1/137 (0.7%) (*h* = −0.30); violent 27/655 (4.1%) versus 0/137 (0%) (*h* = −0.41). Orofacial/vocal features (any; denominator all events) were 174/717 (24.3%) in iRBD versus 8/140 (5.7%) in NT1-RBD (*h* = −0.55) (see Supplementary Tables S2, S3). Direct visual comparison of event-level semiology is shown in Figure 2, and event-level topography in Figure 4. Event-duration distributions were heavy-tailed and similar between groups (Mann–Whitney *p* = 0.383) (see Supplementary Figure S5).

### 3.7 Robustness analyses

Two prespecified checks corroborated the primary patterns. First, patient-label permutation tests (10,000 permutations) yielded empirical *p* = 0.0001 for upper-limb involvement and *p* = 0.0083 for complex behaviour (Supplementary Table S5 and Supplementary Figure S4a,b). Second, Beta-Binomial models (Beta [1,1] priors) yielded posterior means (95% CrI) of 73.5% (70.1–76.7%) in iRBD versus 22.9% (16.2–30.7%) in NT1-RBD for upper-limb involvement, and 19.8% (16.9–23.1%) versus 5.8% (2.6–11.2%) for complex behaviour (Supplementary Table S4). Permutation tests shuffled labels at the patient level and recomputed the event-level difference at each shuffle; they therefore corroborate event-level separation under label-exchangeability, while the patient remains the declared primary unit of inference.

### 3.8 Antidepressant-negative sensitivity

In patients not taking antidepressants, the direction of group differences was preserved but the dominant-region contrast attenuated: dominant lower-limb in NT1-RBD 13/14 (92.9%) vs. iRBD 15/18 (83.3%) (Fisher *p* = 0.6128; Cohen’s *h* = 0.30). Complexity and violent features remained strongly separative [any complex: iRBD 14/18 (77.8%) vs. NT1-RBD 2/14 (14.3%), *p* = 0.0010, *h* = −1.38; violent: 8/18 (44.4%) vs. 0/14 (0%), *p* = 0.0044, *h* = −1.46]. These findings indicate that the core semiological dissociation, complex/affective content, is not explained by antidepressant exposure, while the dominant-region separation is smaller and imprecise in this subset. This attenuation was expected given the markedly reduced n and fewer events per patient in the subset; importantly, complex and violent features remained strongly separative.

### 3.9 Matched-subset sensitivity (effect-size focus)

We repeated patient-level contrasts in two under-powered but informative subsets. First, restricting iRBD to the 12 youngest patients (no age overlap with NT1-RBD) did not alter the large categorical separation (e.g., dominant lower 78.3% vs. 25.0%; dominant upper 8.7% vs. 75.0%; any complex 13.0% vs. 75.0%; |*h*| ≈ 1.0–1.7). Second, in male-only analyses (iRBD 31; NT1-RBD 8) the dissociation was at least as large (|*h*| ≈ 1.1–2.2), with the per-patient complex-event proportion remaining higher in iRBD (Mann–Whitney *p* < 0.001; Cliff’s *δ* ≈ −0.77). Full tables appear in Supplementary Sensitivity.

### 3.10 Sex-stratified descriptive contrasts

The analytic cohort was male-predominant in iRBD (31 male/3 female) and female-predominant in NT1-RBD (8 male/15 female). Directionality of group differences was consistent within both sexes, but estimates in females are imprecise due to the small iRBD female *n* (3). See Supplementary Table S8 for sex-split “ever” features (dominant region, complex, violent, scenic).

## 4 Discussion

This study identifies a reproducible semiological dissociation between isolated REM sleep behaviour disorder (iRBD) and narcolepsy type 1 with REM sleep behaviour disorder (NT1-RBD): iRBD shows predominance of upper-limb involvement with a higher burden of complex, scenic and violent dream-enactment, whereas NT1-RBD preferentially recruits the lower limbs (see Figures 1–4). The separation is large at the patient level (Cohen’s *h* ≈ 0.5–0.9 across key features) and persists across matched-subset sensitivities (youngest iRBD; male-only), and across event-level permutation tests that preserve within-patient clustering. The pattern remained after excluding antidepressant-exposed patients for the content-complexity features, although the dominant-region contrast attenuated in that subset, which we report transparently. These data extend a body of work establishing iRBD as a prodromal synucleinopathy in most patients, with long-horizon phenoconversion rates exceeding 70–90% at 10–14 years (21, 22).

Mechanistically, the results align with contemporary models of REM-atonia circuitry that place a glutamatergic “REM-on” generator in the pontine sublaterodorsal (SLD/subcoeruleus) region projecting to medullary premotor populations that impose glycine/GABA-mediated hyperpolarisation on spinal motor pools (23–25). In humans and rodents, interference with these medullary inhibitory neurons, particularly in the ventromedial medulla, yields REM sleep without atonia accompanied by vigorous, often violent movements, an experimental facsimile of RBD (26). Our finding that iRBD behaviours are richer in complex and scenic content, with prominent upper-limb expression, is consistent with disruption of descending reticulospinal control and cortical–subcortical integration during REM sleep, rather than a mere failure of static tone suppression. Conversely, the lower-limb bias in NT1-RBD suggests a different balance of network vulnerability and motor pattern recruitment during REM sleep, a motif echoed by the lower-limb predisposition of cataplexy outside REM sleep. We deliberately keep orexin speculation to a minimum; it suffices to note that NT1 is defined by loss of hypothalamic orexin neurons and/or very low CSF orexin-A concentrations (27, 28), with wide-ranging projections to brainstem arousal nuclei and premotor systems (29). Our data show that, whatever the upstream transmitter deficit, the downstream semiology in NT1-RBD is not a carbon copy of iRBD.

Methodologically, two design choices are central. First, we declared the patient as the primary unit of inference and report “ever” features with effect-size emphasis. Because the iRBD cohort contributed more events, we complemented “ever” with the per-patient proportion of complex body-involving events, which reproduced the between-group separation. Second, to avoid extrapolation across non-overlap, we eschewed conventional covariate adjustment and bounded confounding through pre-specified sensitivities (age-restricted; male-only), where the direction and magnitude of effects were conserved despite reduced power. Permutation tests that shuffled patient labels while preserving event clustering offered convergent support at the event level.

The present findings sharpen the nosological distinction between iRBD and NT1-RBD. Clinically, limb-topography and event complexity are features that can be recognised at the bedside and by expert video-review, and could help triage patients into surveillance pathways and trials. For iRBD, the predominance of complex/violent enactments aligns with a literature linking the syndrome to progressive synucleinopathy (21, 22, 30) and to selective vulnerability of REM-atonia circuitry (25, 26). For NT1-RBD, the semiology calls for targeted physiology: REM-state modulation of postural and locomotor generators, the coupling between phasic twitches and gross movements, and orexin-independent contributions of basal forebrain and medullary networks, questions directly testable with concurrent high-density EMG and brainstem-sensitive neuroimaging.

Several major limitations deserve care. The groups differ markedly in age and sex; although effect sizes remained large in matched-subset analyses, residual confounding cannot be excluded. Antidepressant exposure was handled by exclusion and sensitivity analysis; some residual influence on content cannot be ruled out. “Ever” outcomes are opportunity-sensitive; we mitigated this with a per-patient proportion and by placing the patient-level unit at the centre of inference, but richer multilevel modelling will be valuable as larger, overlapping cohorts accrue. Finally, our work is descriptive by design; causal links between specific circuit lesions and limb-topography await invasive physiology and longitudinal imaging. PLMs are another potential confound. PLMI was available only for a subset of iRBD patients and not systematically for NT1-RBD in this clinical epoch. Because PLMs generally increase with age, any residual PLM-related lower-limb activity would be expected to bias the older iRBD group toward lower-limb involvement and thereby attenuate, not create, the lower-limb-weighted phenotype observed in NT1-RBD. Our *a priori* exclusions of events adjacent to arousals and respiratory events, and our emphasis on patient-level inference, partly mitigate this concern, but future studies with systematic PLM scoring in both groups are needed.

In conclusion, semiology during REM sleep carries anatomy-in-motion. The present dissociation, upper-limb, complex enactments in iRBD versus lower-limb-weighted behaviours in NT1-RBD, offers a compact clinical read-out of distinct network liabilities during REM sleep. It invites mechanistic experiments that move beyond monolithic “loss of atonia” models toward a circuit-level account of how dream content couples to distributed motor programs in disease (8, 20, 25, 26, 30, 31).

## Data Availability

The data analyzed in this study is subject to the following licenses/restrictions: The dataset contains sensitive clinical information and, under GDPR and local data protection regulations, cannot be shared in identifiable form; access to the underlying pseudonymised patient-level data can only be granted upon reasonable request to the corresponding author and would require prior approval by the institutional ethics committee and data protection officer. Requests to access these datasets should be directed to the corresponding author.
