# Supplementary material for: Differential motor signatures in isolated and narcolepsy-related REM sleep behaviour disorder: a preliminary study
Source: Front Neurol. 2026 Jan 14;16:1749306. doi: 10.3389/fneur.2025.1749306 (PMC12847386; doi:10.3389/fneur.2025.1749306)
Supplement: Supplementary file 1 [file Supplementary_file_1.docx]

# **Supplement to:**

**Differential Motor Signatures in Isolated and Narcolepsy-related REM Sleep Behavior Disorder: A Preliminary Study**

This supplement includes additional statistical notes, effect size tables, and supporting figures for the associated *original research*.

**Supplementary Contents**

1. Demographic and Sleep Architecture Data
2. Supplementary Tables and Figures
3. Semiology of RBD Events
4. References

**1 Demographics and Polysomnography Data**

**Supplementary Table S1.** Sociodemographic and Polysomnography Data of Primary (iRBD) and Secondary (NT1 with RBD) RBD patients.

Counts are n (%). Between‑group p‑values are Fisher’s exact; effect size is Cohen’s h for the male proportion (NT1‑RBD minus iRBD).

| **Characteristic** | **iRBD (n=34)** | **NT1‑RBD (n=23)** | **Fisher p** | **Cohen’s h (male)** |
| --- | --- | --- | --- | --- |
| Sex — Male | 31 (91.2%) | 8 (34.8%) | <0.0001 | -1.277 |
| Sex — Female | 3 (8.8%) | 15 (65.2%) | <0.0001 | -1.277 |
| Antidepressant — Yes | 11 (32.4%) | 8 (34.8%) | 1.0000 |  |
| Antidepressant — No | 23 (67.6%) | 15 (65.2%) | 1.0000 |  |

Note: Cohen’s h column reports the effect size for the male proportion only; the same value is shown on both sex rows for convenience.

| **Variable** | **iRBD Mean** | **iRBD SD** | **NT1 Mean** | **NT1 SD** | **p** | **Cliff δ (NT1−iRBD)** |
| --- | --- | --- | --- | --- | --- | --- |
| Age | 69.03 | 6.83 | 28.78 | 9.09 | <0.0001 | -1.000 |
| BMI | 26.51 | 2.83 | 28.30 | 6.28 | 0.3369 | 0.152 |
| TST | 326.21 | 53.95 | 433.52 | 94.73 | <0.0001 | 0.781 |
| WASO | 108.59 | 60.97 | 71.39 | 62.93 | 0.0064 | -0.430 |
| SL | 33.88 | 63.38 | 7.26 | 8.38 | <0.0001 | -0.715 |
| SE | 70.85 | 13.19 | 84.45 | 12.45 | <0.0001 | 0.683 |
| RL (min) | 148.90 | 110.97 | 37.39 | 59.67 | <0.0001 | -0.816 |
| AHI | 11.78 | 14.60 | 2.73 | 4.82 | 0.0010 | -0.518 |
| AI (events/hr) | 26.29 | 14.37 | 20.98 | 10.29 | 0.1744 | -0.215 |
| PLMI (e/hr) | 28.8 | 40.4 | n/a | n/a | n/a | n/a |
| N1% | 21.31 | 11.45 | 14.30 | 7.14 | 0.0209 | -0.364 |
| N2% | 39.30 | 8.66 | 39.57 | 8.78 | 0.8072 | 0.040 |
| N3% | 19.57 | 9.66 | 24.02 | 5.49 | 0.0366 | 0.330 |
| REM% | 19.31 | 6.14 | 22.10 | 6.48 | 0.1004 | 0.260 |
| RBD duration (s) | 14.21 | 18.94 | 18.54 | 30.79 | 0.3826 | 0.047 |

***Abbreviations***: %, percentage; AI, arousal index; AHI, apnoea/hypopnoea index; BMI, body mass index; iRBD, isolated REM Behaviour Disorder; n/a, not-available; N, number; N1-3, non-REM sleep stages one to three; NREM, non-rapid eye movement sleep; NT1, narcolepsy type 1; PLMI, periodic limb movement index; REM, rapid eye movement; SE, sleep efficiency; SL, sleep latency; TST, total sleep time; WASO, wakefulness after sleep onset. PLMI was not systematically scored for NT1-RBD in the analytic cohort; values are therefore reported descriptively for iRBD only and not compared statistically.

**1.1 Supplementary Note**

Unit of inference: the patient is the primary unit of analysis. Event‑level summaries are provided as counts, proportions, and effect sizes but without p‑values, to avoid pseudo‑replication from clustered events.

Denominators: topography and orofacial/vocal features use all scored events; complexity/content qualifiers (complex, scenic, violent, self‑referential) use only body‑involvement events. Topographical categories are non‑exclusive and may sum to >100%.

Effect sizes. Binary contrasts are quantified as Cohen’s h (arcsine‑difference metric), with conventional thresholds (≈0.2 small, 0.5 medium, 0.8 large). Distributions of per‑patient proportions (e.g., proportion complex) are compared using the Mann–Whitney U test with Cliff’s δ as effect size. For Cliff’s δ we report bootstrap 95% CIs (5,000 resamples). For proportions we provide Bayesian Beta–Binomial posterior means with 95% credible intervals under Beta(1,1) priors for prespecified features.

Separation and modelling stance: several features exhibit perfect/near‑perfect separation between groups. Because age overlap is minimal, conventional adjusted logistic models entail extrapolation. Accordingly, primary inference relies on exact tests and effect sizes at the patient level. As sensitivity checks, we report (i) patient‑label permutation tests (10,000 permutations; preserving within‑patient clustering) for two headline features and (ii) Bayesian Beta–Binomial estimation for upper‑limb involvement and complex behaviour. Event‑duration comparisons are descriptive at the event level (Mann–Whitney and Cliff’s δ provided for transparency).

**2 Supplementary Tables and Figures**

All analyses below refer to the analytic cohort comprising isolated RBD (iRBD) = 34 patients and NT1‑RBD = 23 patients, and to the scored event corpus (iRBD = 717 events; NT1‑RBD = 140 events; body‑involvement events: iRBD = 655; NT1‑RBD = 137).

## Supplementary Table S2. Event‑level counts (descriptive only)

| **Feature (event‑level)** | **NT1 n** | **NT1 total** | **NT1 %** | **iRBD n** | **iRBD total** | **iRBD %** |
| --- | --- | --- | --- | --- | --- | --- |
| RBD with body involvement | 137 | 140 | 97.9 | 655 | 717 | 91.4 |
| Solely vocal/orofacial | 0 | 140 | 0.0 | 61 | 717 | 8.5 |
| Orofacial and/or vocal (any) | 8 | 140 | 5.7 | 174 | 717 | 24.3 |
| Self-oriented (body-only) | 1 | 137 | 0.7 | 35 | 655 | 5.3 |
| Scenic (body-only) | 9 | 137 | 6.6 | 111 | 655 | 16.9 |
| Violent (body-only) | 0 | 137 | 0.0 | 27 | 655 | 4.1 |
| Complex (body-only) | 8 | 137 | 5.8 | 130 | 655 | 19.8 |
| Elementary (body-only) | 129 | 137 | 94.2 | 525 | 655 | 80.2 |
| Head & neck only | 1 | 140 | 0.7 | 19 | 717 | 2.6 |
| Upper limbs (any) | 32 | 140 | 22.9 | 527 | 717 | 73.5 |
| Head & neck (any) | 22 | 140 | 15.7 | 131 | 717 | 18.3 |
| Trunk (any) | 47 | 140 | 33.6 | 32 | 717 | 4.5 |
| Lower limbs (any) | 125 | 140 | 89.3 | 288 | 717 | 40.2 |

*Denominators: rows marked '(body‑only)' use body‑involvement totals (iRBD=655; NT1‑RBD=137). Others use all events (iRBD=717; NT1‑RBD=140). Topographical categories are non‑exclusive. Laterality scored: iRBD 636/717; NT1-RBD 137/140; among scored: bilateral 368/636 vs 97/137; unilateral 268/636 vs 40/137.*

## Supplementary Table S3. Event‑level effect sizes (Cohen’s h)

| **Feature** | **NT1 n** | **NT1 total** | **iRBD n** | **iRBD total** | **NT1 %** | **iRBD %** | **Cohen’s h (NT1 − iRBD)** |
| --- | --- | --- | --- | --- | --- | --- | --- |
| RBD with body involvement | 137 | 140 | 655 | 717 | 97.9 | 91.4 | 0.30 |
| Solely vocal/orofacial | 0 | 140 | 61 | 717 | 0.0 | 8.5 | -0.59 |
| Orofacial and/or vocal (any) | 8 | 140 | 174 | 717 | 5.7 | 24.3 | -0.55 |
| Self-oriented (body-only) | 1 | 137 | 35 | 655 | 0.7 | 5.3 | -0.30 |
| Scenic (body-only) | 9 | 137 | 111 | 655 | 6.6 | 16.9 | -0.33 |
| Violent (body-only) | 0 | 137 | 27 | 655 | 0.0 | 4.1 | -0.41 |
| Complex (body-only) | 8 | 137 | 130 | 655 | 5.8 | 19.8 | -0.44 |
| Elementary (body-only) | 129 | 137 | 525 | 655 | 94.2 | 80.2 | 0.44 |
| Head & neck only | 1 | 140 | 19 | 717 | 0.7 | 2.6 | -0.16 |
| Upper limbs (any) | 32 | 140 | 527 | 717 | 22.9 | 73.5 | -1.06 |
| Head & neck (any) | 22 | 140 | 131 | 717 | 15.7 | 18.3 | -0.07 |
| Trunk (any) | 47 | 140 | 32 | 717 | 33.6 | 4.5 | 0.81 |
| Lower limbs (any) | 125 | 140 | 288 | 717 | 89.3 | 40.2 | 1.10 |

*Positive values indicate higher prevalence in NT1‑RBD; negative values indicate higher prevalence in iRBD.*

## Supplementary Table S4. Bayesian posterior estimates (Beta–Binomial, Beta[1,1])

| **Group** | **Feature** | **Posterior mean** | **95% CrI lower** | **95% CrI upper** |
| --- | --- | --- | --- | --- |
| NT1-RBD | Upper limb involvement (any) | 0.2324 | 0.1669 | 0.3050 |
| iRBD | Upper limb involvement (any) | 0.7344 | 0.7015 | 0.7660 |
| NT1-RBD | Complex behaviour (body-only) | 0.0647 | 0.0303 | 0.1110 |
| iRBD | Complex behaviour (body-only) | 0.1994 | 0.1697 | 0.2308 |

## Supplementary Table S5. Patient‑label permutation tests (10,000 permutations)

| **Feature** | **Observed difference (pp)** | **Permutation p (two‑sided)** |
| --- | --- | --- |
| Upper limb involvement (any; all events) | -50.6 | 0.0001 |
| Complex behaviour (body-only) | -14.0 | 0.0083 |

## Supplementary Table S6. Patient‑level prevalence of key REM‑behaviour features ('ever')

| **Feature (patient‑level 'ever')** | **iRBD n/N (%)** | **NT1 n/N (%)** | **Cohen’s h (NT1 − iRBD)** | **Fisher p** |
| --- | --- | --- | --- | --- |
| Upper-limb involvement | 33/34 (97.1%) | 11/23 (47.8%) | -1.2696 | <0.00001 |
| Lower-limb involvement | 30/34 (88.2%) | 22/23 (95.7%) | 0.2801 | 0.6384 |
| Trunk involvement | 14/34 (41.2%) | 11/23 (47.8%) | 0.1339 | 0.7862 |
| Head/neck involvement | 26/34 (76.5%) | 9/23 (39.1%) | -0.7770 | 0.0062 |
| Any complex event | 27/34 (79.4%) | 3/23 (13.0%) | -1.4607 | <0.00001 |
| Scenic | 23/34 (67.6%) | 3/23 (13.0%) | -1.1925 | <0.00001 |
| Violent | 16/34 (47.1%) | 0/23 (0.0%) | -1.5119 | <0.00001 |
| Self-referential | 13/34 (38.2%) | 1/23 (4.3%) | -0.9132 | 0.0041 |
| Vocal/orofacial (any) | 27/34 (79.4%) | 3/23 (13.0%) | -1.4607 | <0.00001 |
| **Bilateral lower-limb** | **29/34 (85.3%)** | **20/23 (87.0%)** | **0.0481** | **1.0000** |
| Dominant region = lower | 7/34 (20.6%) | 18/23 (78.3%) | 1.2296 | <0.00001 |
| Dominant region = upper | 24/34 (70.6%) | 2/23 (8.7%) | -1.3965 | <0.00001 |

## Supplementary Table S7. Per‑patient proportion of complex events

| **Group** | **Median** | **IQR** | **Mann–Whitney p** | **Cliff’s δ [95% CI]** |
| --- | --- | --- | --- | --- |
| iRBD | 0.21 | 0.05–0.33 | 0.000008 | -0.665 [-0.853, -0.441] |
| NT1‑RBD | 0.00 | 0.00–0.00 |  |  |

*Denominator per patient: number of body‑involvement events. Cliff’s δ is computed as NT1 − iRBD (negative indicates higher in iRBD).*

# Supplementary Table S8. Sex‑stratified patient‑level descriptive contrasts (analytic cohort)

Counts are n/N (%). ‘Ever’ features are computed at the patient level from the event corpus using the same rules as Table S6 (complex/content qualifiers use body‑involvement events; topography uses all events). Fisher’s exact p is provided for orientation only; precision is limited by small cell sizes, especially in iRBD females (n=3).

Sample sizes by sex: iRBD — M=31, F=3; NT1‑RBD — M=8, F=15.

| **Feature** | **Sex** | **iRBD n/N (%)** | **NT1 n/N (%)** | **Fisher p (descriptive)** |
| --- | --- | --- | --- | --- |
| Dominant region = lower | M | 7/31 (22.6%) | 6/8 (75.0%) | 0.0098 |
| Dominant region = lower | F | 0/3 (0.0%) | 12/15 (80.0%) | 0.0245 |
| Dominant region = upper | M | 21/31 (67.7%) | 0/8 (0.0%) | 0.0007 |
| Dominant region = upper | F | 3/3 (100.0%) | 2/15 (13.3%) | 0.0123 |
| Any complex event | M | 24/31 (77.4%) | 0/8 (0.0%) | 0.0001 |
| Any complex event | F | 3/3 (100.0%) | 3/15 (20.0%) | 0.0245 |
| Violent | M | 14/31 (45.2%) | 0/8 (0.0%) | 0.0337 |
| Violent | F | 2/3 (66.7%) | 0/15 (0.0%) | 0.0196 |
| Scenic | M | 20/31 (64.5%) | 0/8 (0.0%) | 0.0012 |
| Scenic | F | 3/3 (100.0%) | 3/15 (20.0%) | 0.0245 |

# **Supplementary Sensitivity — Matched Subsets (Effect‑size focus)**

We present two matched‑subset sensitivities to bound confounding: (A) an age‑restricted iRBD subset (youngest iRBD; still no age overlap) and (B) a male‑only contrast. In both, inference remains patient‑level; effect sizes are primary; p‑values are descriptive.

## A) Age‑restricted iRBD (youngest iRBD vs all NT1‑RBD)

Rationale: iRBD is markedly older than NT1‑RBD. Because no age overlap exists (youngest iRBD 56 y; oldest NT1‑RBD 52 y), we restrict iRBD to the 12 youngest patients (median 61.0 [IQR 59.0–64.2] years) and compare with all NT1‑RBD (n=23; median 26.2 [IQR 23.5–36.3] years).

Patient‑level 'ever' features:

| **Feature** | **iRBD n/N (%)** | **NT1 n/N (%)** | **Cohen's h (NT1−iRBD)** | **Fisher p (2‑sided)** |
| --- | --- | --- | --- | --- |
| Dominant region = lower | 3/12 (25.0%) | 18/23 (78.3%) | 1.124 | 0.0038 |
| Dominant region = upper | 9/12 (75.0%) | 2/23 (8.7%) | -1.496 | 0.0001 |
| Any complex event (body-only) | 9/12 (75.0%) | 3/23 (13.0%) | -1.355 | 0.0005 |
| Violent (body-only) | 7/12 (58.3%) | 0/23 (0.0%) | -1.738 | 0.0001 |
| Scenic (body-only) | 7/12 (58.3%) | 3/23 (13.0%) | -0.999 | 0.0146 |
| Upper-limb involvement | 11/12 (91.7%) | 11/23 (47.8%) | -1.029 | 0.0132 |
| Head/neck involvement | 10/12 (83.3%) | 9/23 (39.1%) | -0.949 | 0.0298 |

Per‑patient complex proportion: iRBD median 0.16 [0.02–0.34] vs NT1‑RBD 0.00 [0.00–0.00]; Mann–Whitney p=0.0004; Cliff’s δ (NT1−iRBD) = -0.627.

## B) Male‑only (iRBD males vs NT1‑RBD males)

Sample sizes: iRBD 31 males (median age 68.5 y); NT1‑RBD 8 males (median age 25.2 y).

| **Feature** | **iRBD n/N (%)** | **NT1 n/N (%)** | **Cohen's h (NT1−iRBD)** | **Fisher p (2‑sided)** |
| --- | --- | --- | --- | --- |
| Dominant region = lower | 7/31 (22.6%) | 6/8 (75.0%) | 1.104 | 0.0098 |
| Dominant region = upper | 21/31 (67.7%) | 0/8 (0.0%) | -1.934 | 0.0007 |
| Any complex event (body-only) | 24/31 (77.4%) | 0/8 (0.0%) | -2.151 | 0.0001 |
| Violent (body-only) | 14/31 (45.2%) | 0/8 (0.0%) | -1.474 | 0.0337 |
| Scenic (body-only) | 20/31 (64.5%) | 0/8 (0.0%) | -1.865 | 0.0012 |
| Upper-limb involvement | 30/31 (96.8%) | 3/8 (37.5%) | -1.462 | 0.0005 |
| Head/neck involvement | 23/31 (74.2%) | 4/8 (50.0%) | -0.505 | 0.2206 |

Per‑patient complex proportion (male‑only): iRBD median 0.21 [0.03–0.33] vs NT1‑RBD 0.00 [0.00–0.00]; Mann–Whitney p=0.0006; Cliff’s δ (NT1−iRBD) = -0.774.

# **Sensitivity Analysis — Bayesian Hierarchical Model (Beta–Binomial)**

We fit a patient‑level Beta–Binomial regression to the number of complex events out of body‑involvement events (yᵢ ~ BetaBinomial(nᵢ, μᵢ, κ)). The mean parameter used a logit link: logit(μᵢ) = β₀ + β₁·I(NT1‑RBD) + β₂·I(male). Priors were weakly informative: βⱼ ~ Normal(0, 2.5²) and log κ ~ Normal(0, 1²). We used MAP estimation with a Laplace (normal) posterior approximation and report effect sizes and 95% credible intervals. This model is explicitly a sensitivity analysis; the primary unit of inference remains the patient‑level exact tests and effect‑size summaries.

| **Parameter** | **Posterior mean** | **SD** | **2.5%** | **97.5%** |
| --- | --- | --- | --- | --- |
| Intercept | -0.805 | 0.151 | -1.104 | -0.511 |
| NT1 (vs iRBD) | -2.163 | 0.360 | -2.874 | -1.454 |
| Male (vs female) | -0.619 | 0.147 | -0.906 | -0.331 |
| Concentration κ | 5.60 | 0.43 | 4.79 | 6.50 |

Posterior predictive complex‑event proportions (marginalised by observed sex mix):

| **Group** | **Posterior mean μ** | **SD** | **2.5%** | **97.5%** |
| --- | --- | --- | --- | --- |
| iRBD | 0.205 | 0.014 | 0.178 | 0.234 |
| NT1‑RBD | 0.046 | 0.021 | 0.017 | 0.099 |

Difference in predicted complex proportion (NT1 − iRBD): mean -0.159, 95% CrI [-0.187, -0.120]. Negative values favour iRBD (higher complex‑event proportion).


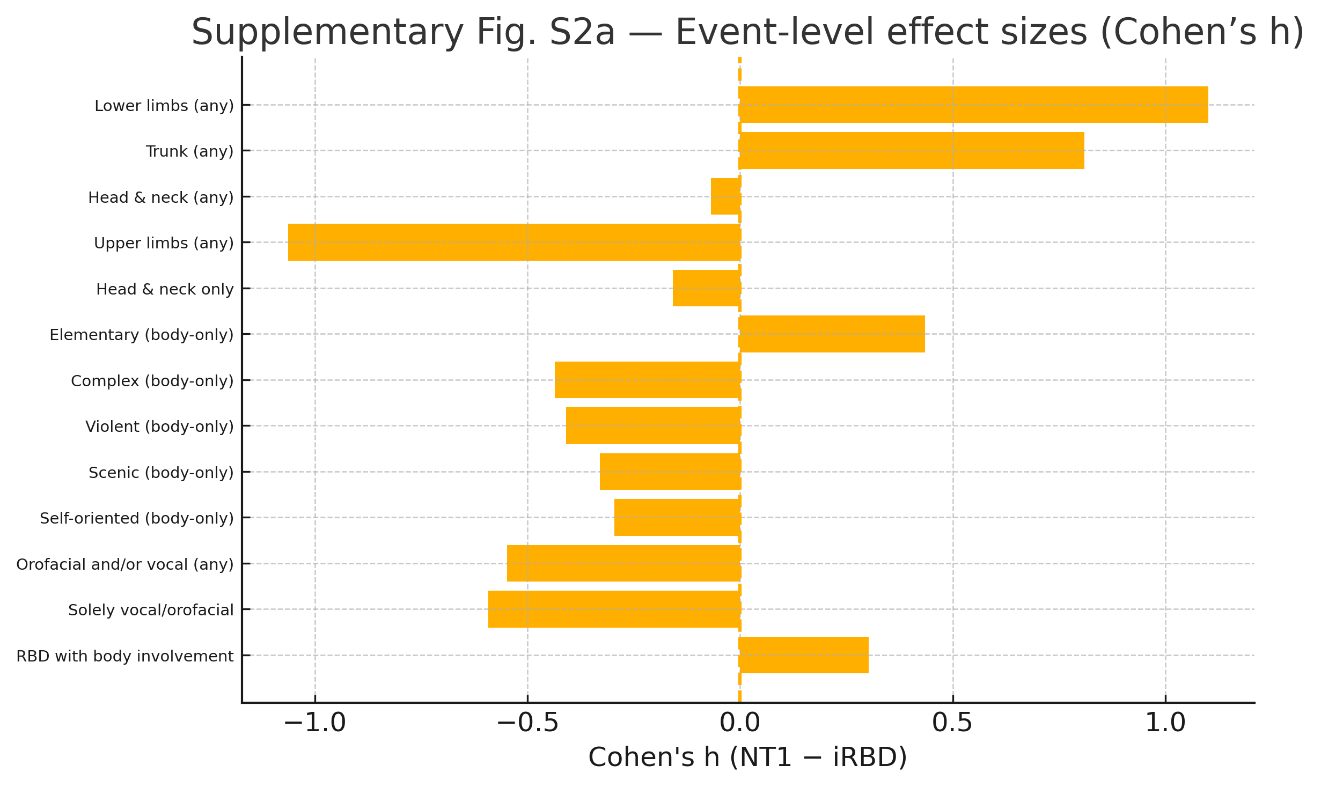
Supplementary Fig. S1a. Event‑level effect sizes (Cohen’s h).

*Bars show Cohen’s h (NT1 − iRBD) using denominators specified in Table S2. Vertical dashed line marks zero.*

Supplementary Fig. S1b. Topography distributions by group (denominator = all events).


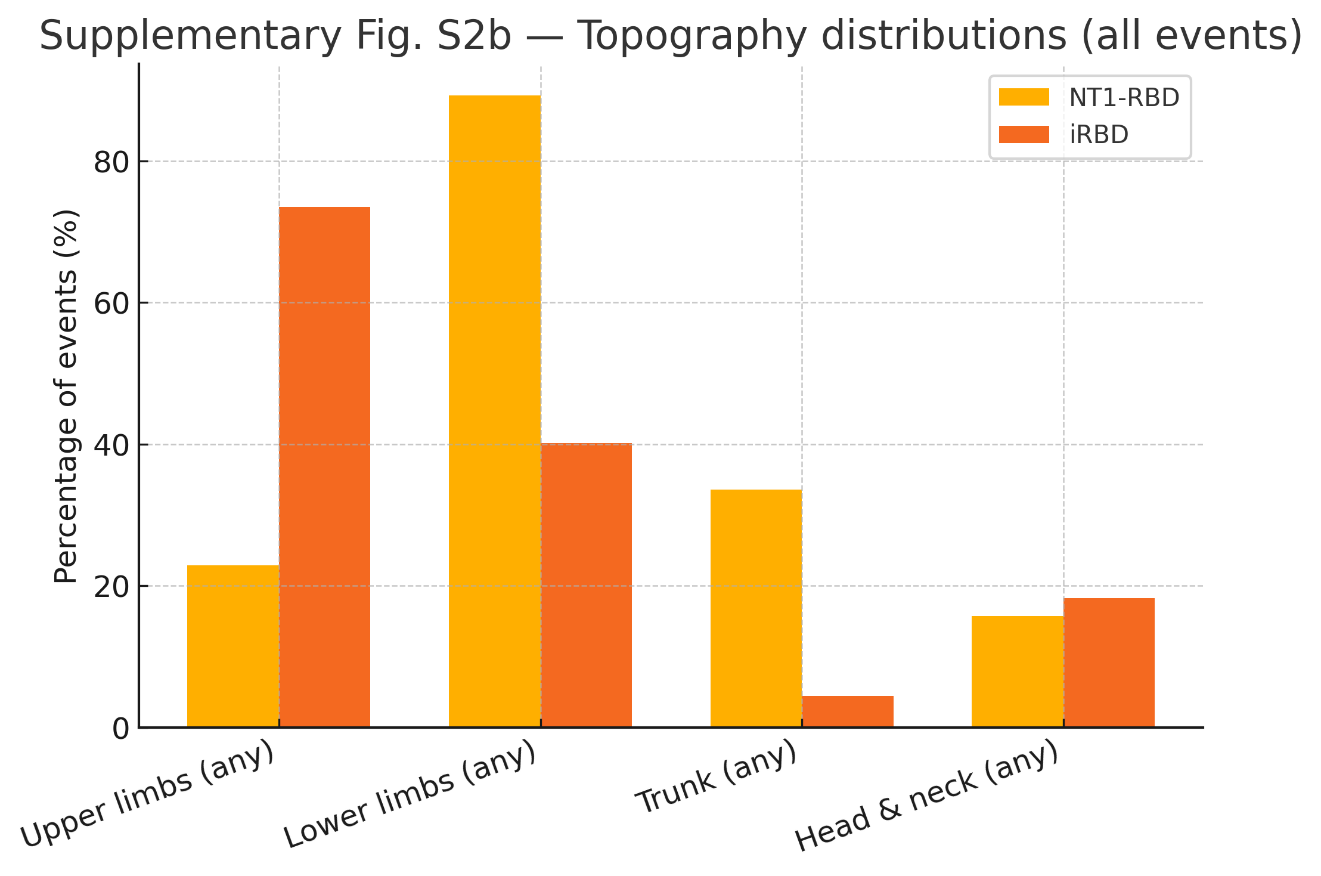


*Percentages computed from all scored events (iRBD = 717; NT1‑RBD = 140).* *Topographical categories are non‑exclusive; percentages may sum to >100%.*


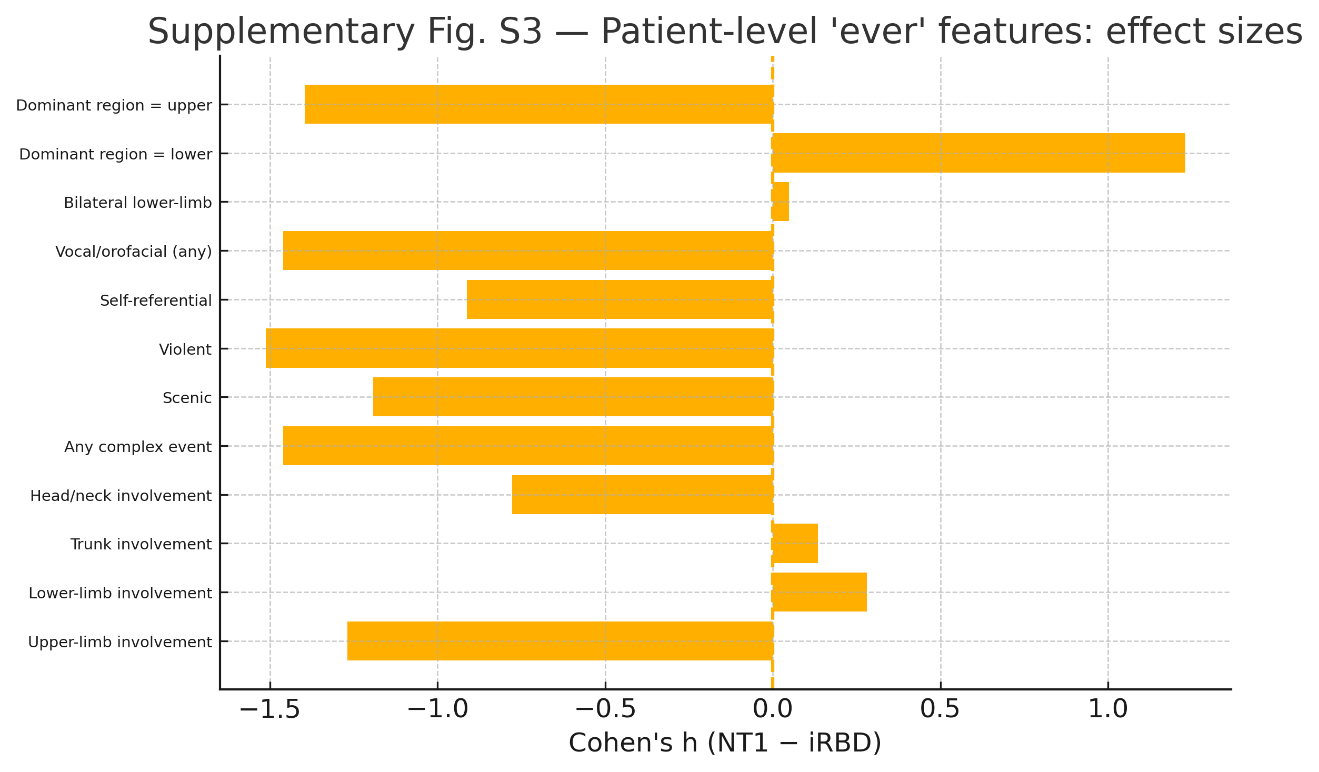
Supplementary Fig. S2. Patient‑level 'ever' features: effect sizes (Cohen’s h).

*Cohen’s h calculated from the patient‑level ‘ever’ table (S6). Negative values favour iRBD. Topographical categories are non‑exclusive; percentages may sum to >100%.*


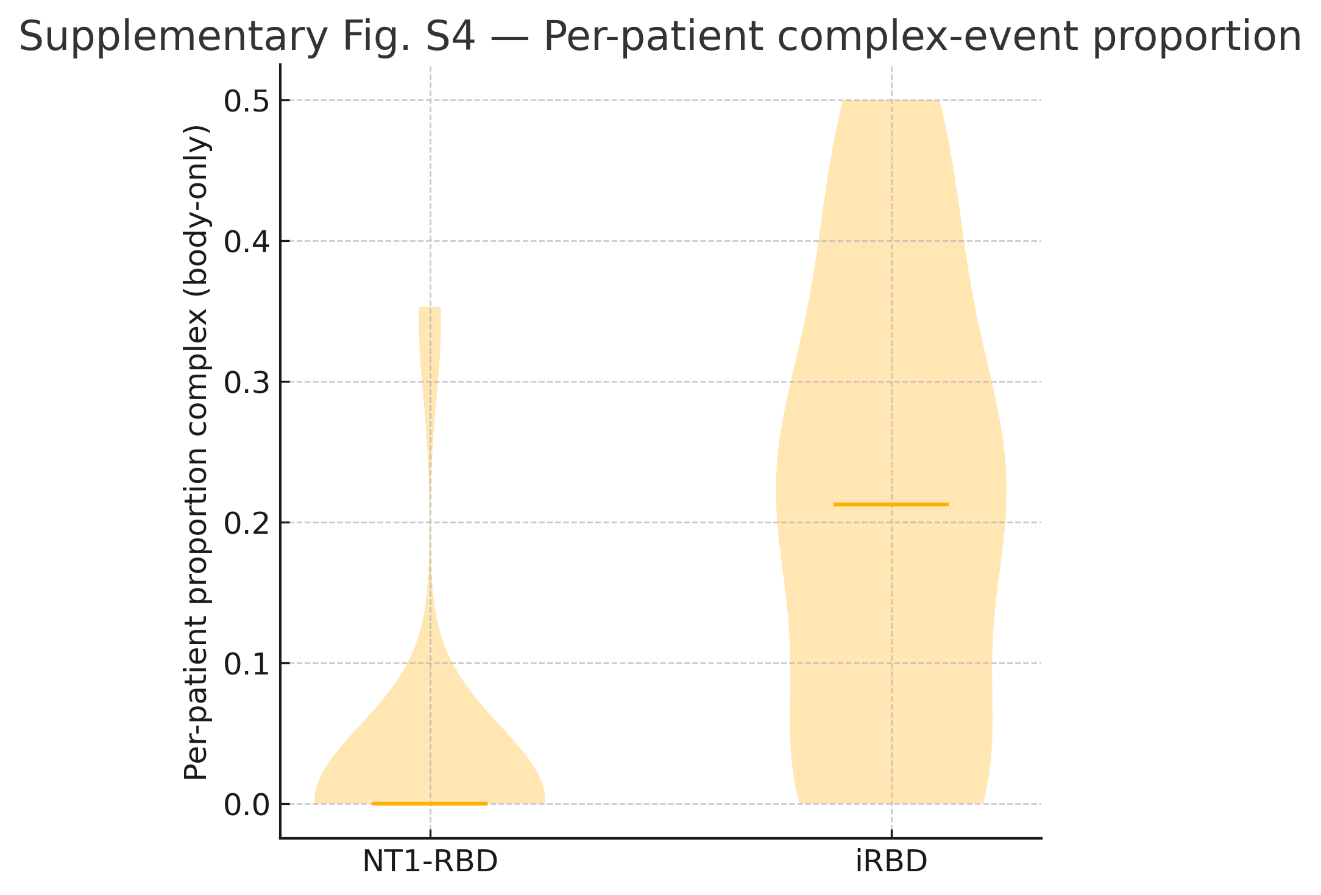


Supplementary Fig. S3. Per‑patient complex‑event proportion by group (violin).

*Violins show the distribution of per‑patient proportion complex (denominator: body‑involvement events). Median line shown.*


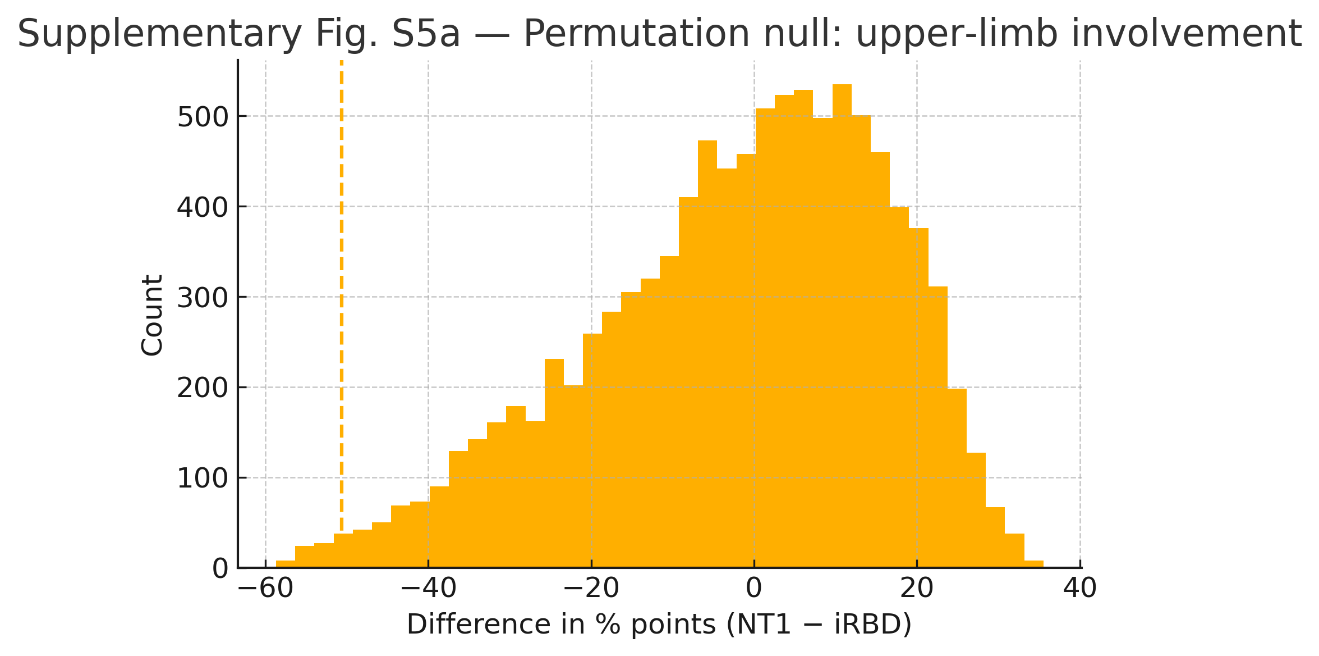


Supplementary Fig. S4a. Permutation null for upper‑limb involvement (any).

*Histogram of differences from 10,000 patient‑label permutations; vertical line = observed difference. See Table S5 for p‑value.*


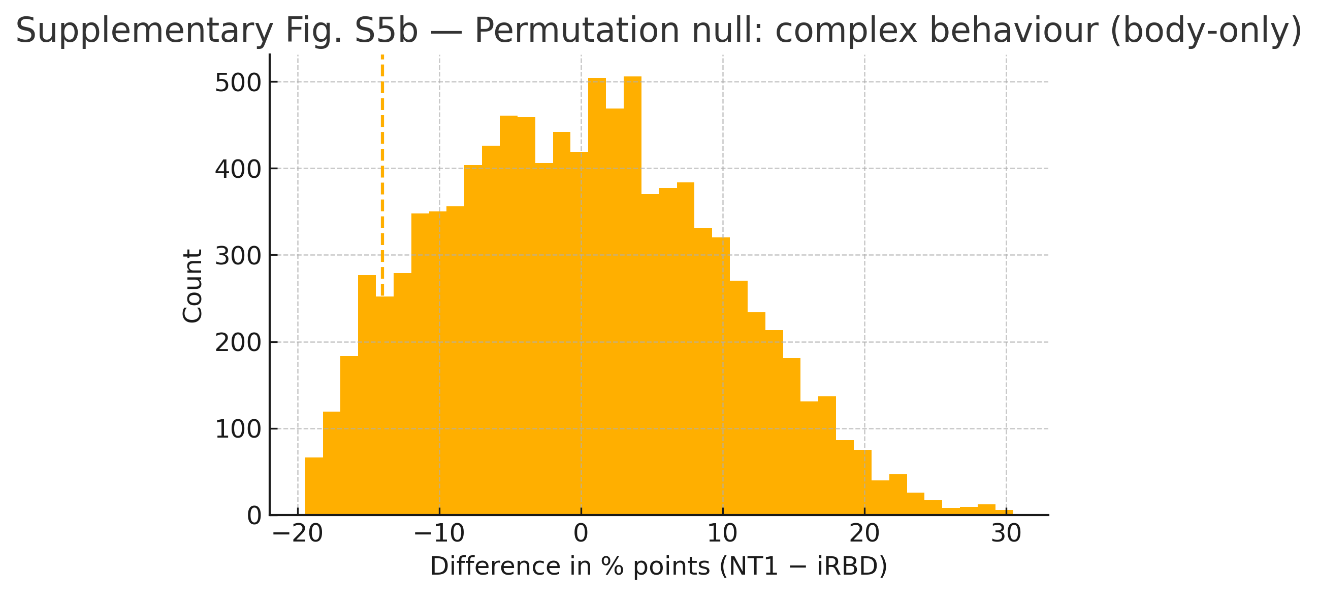


Supplementary Fig. S4b. Permutation null for complex behaviour (body‑only).

*Histogram of differences from 10,000 patient‑label permutations; vertical line = observed difference. See Table S5 for p‑value.*


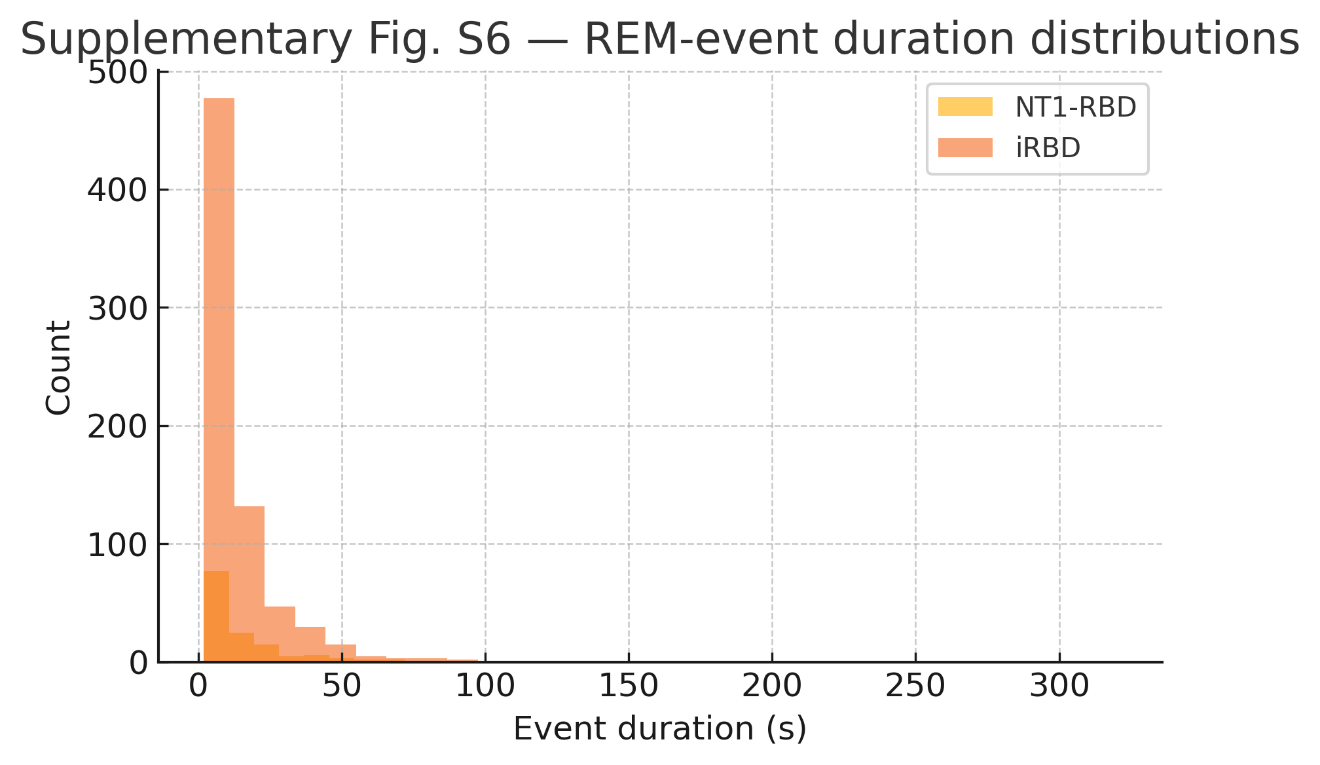


Supplementary Fig. S5. REM sleep‑event duration distributions (event‑level).

*Histogram of event durations per group. Event‑level comparisons are descriptive; patient‑level inference remains primary.*

**3 Semiology of RBD Events**

**Supplementary Table S9. Clinical profile of semiology and motor patterns of RBD events, as previously published[1].**

| Type of Movement | | | | Semiology Description *(method adapted from[2; 3; 4])* |
| --- | --- | --- | --- | --- |
| *Myoclonic events* | | | | sudden, brief, jerky, involuntary movements involving extremities, face and trunk[5] (Fahn et al., 1986). |
| *Simple motor events* | | | | small-amplitude movements (commonly unnoticeable by a bed partner). |
| *Stereotypes* | | | | repetitive, invariant behaviour patterns with no obvious goal or function; automatism-like movements (e.g. smacking, fumbling). |
| *Self-oriented movements* | | | | comprised of touching or rubbing one’s body part. |
| *Complex events* | | Scenic | | *Complex events* were subdivided into *scenic events,* which described apparent “acting out” of dreams and *violent events,* which referred to large-amplitude, potentially injurious movements (e.g. kicking, punching). |
|  |  | *Violent* | |  |
| *Vocalizations* | | | | RBD-related . |
| *Orofacial events* | | | | e.g. clenching, grimacing. |
| *Topographical distribution* | Head/neck | | | Distribution of the motor events was established depending on involved body part(s). Movements were topographically categorized into *head/neck*, *trunk* (rolling and pelvic movements), *upper limbs*, *lower limbs*. |
|  | Trunk | | |  |
|  | Upper limbs | | |  |
|  | Lower limbs | | |  |
| *Spatial distribution* | Focal | | Unilateral | Spatial distribution was subcategorized into *focal* (localized to one body part), *multifocal* (involving non-contiguous body parts), *proximal* and *distal*. *Laterality* of movements was described as *unilateral* or *bilateral*. |
|  | Multifocal | |  |  |
|  | Proximal | | Bilateral |  |
|  | Distal | |  |  |
| *Sagittal plane change* | | | | the movements that involved truncal/lumbar axial muscles across the yz-octant of the sagittal plane of the sleeping body (see main text Figure 1). |

Supplementary Table S9 summarises the semiology taxonomy and operational definitions used for event classification (topography, complexity, qualifiers, spatial distribution, laterality, and truncal sagittal‑plane change). Categories are non‑exclusive for topography. Content qualifiers (scenic, violent, self‑referential) were applied only to body‑involvement events. The taxonomy is reproduced unchanged for transparency and cross‑study comparability.

*References*

[1] D. Wasserman, S. Gullone, I. Duncan, M. Veronese, V. Gnoni, S. Higgins, A. Birdseye, E.C. Gelegen, P.J. Goadsby, K. Ashkan, K. Ray Chaudhuri, G. Tononi, P. Drakatos, and I. Rosenzweig, Restricted truncal sagittal movements of rapid eye movement behaviour disorder. npj Parkinson's Disease 8 (2022) 26.

[2] U. Seneviratne, D. Reutens, and W. D'Souza, Stereotypy of psychogenic nonepileptic seizures: insights from video-EEG monitoring. Epilepsia 51 (2010) 1159-68.

[3] M. Terzaghi, I. Sartori, R. Mai, L. Tassi, S. Francione, F. Cardinale, L. Castana, M. Cossu, G. LoRusso, R. Manni, and L. Nobili, Sleep-related minor motor events in nocturnal frontal lobe epilepsy. Epilepsia 48 (2007) 335-41.

[4] B. Frauscher, V. Gschliesser, E. Brandauer, H. Ulmer, C.M. Peralta, J. Muller, W. Poewe, and B. Hogl, Video analysis of motor events in REM sleep behavior disorder. Mov Disord 22 (2007) 1464-70.

[5] S. Fahn, C.D. Marsden, and M.H. Van Woert, Definition and classification of myoclonus. Adv Neurol 43 (1986) 1-5.
